# Acceptability and Perceived Usefulness of a Digital Gambling Harm Minimization Tool: Cross-Sectional Study

**DOI:** 10.2196/101430

**Published:** 2026-08-07

**Authors:** Dilushi Chandrakumar, Simone N Rodda, Louise Thornton, Sally M Gainsbury

**Affiliations:** 1 Gambling Treatment and Research Clinic, Brain and Mind Centre, School of Psychology, Faculty of Science, The University of Sydney, 94 Mallett St Camperdown, Sydney, New South Wales, 2050, Australia, +61 2 9351 9559; 2 Psychology and Neuroscience, Auckland University of Technology, Auckland, New Zealand; 3 The Matilda Centre for Research in Mental Health and Substance, Faculty of Medicine and Health, The University of Sydney, Sydney, New South Wales, Australia

**Keywords:** digital tool, gambling, informed decision-making, acceptability, harm minimization

## Abstract

**Background:**

Gambling consumers show low uptake and engagement with tools designed to support safer gambling practices and reduce the risk of experiencing gambling-related harms. We used co-design principles to design and develop a digital tool for safer gambling (BetWell) to overcome known barriers to tool uptake, including a focus on gambling problems. BetWell aims to increase awareness of personal gambling expenditure and knowledge of how gambling products function to support informed decision-making about gambling. It was designed based on behavioral change theories and presents a personalized amalgamation of gambling expenditure relative to alternative spend options, and psychoeducational information via a quiz.

**Objective:**

This exploratory study assessed the perceived acceptability and usefulness of the newly designed digital tool and gathered end-user feedback to improve future prototypes. The study explored whether usability, gambling severity, financial well-being, gambling frequency, spend tracking, activity statement use, and the number of accounts held impacted acceptability and perceived usefulness, and the extent to which these factors independently predicted acceptability and perceived usefulness when controlling for life satisfaction, gambling satisfaction, and demographic variables.

**Methods:**

This cross-sectional study recruited 140 gambling consumers (mean 41.3, SD 10.9 years) via the market research panel CRNRSTONE. Participants accessed and engaged with BetWell and completed an online survey to share their perceptions towards the tool.

**Results:**

The overall acceptability (mean 32/40, SD 5.2) and perceived usefulness (mean 20/25, SD 3.9) of the tool were considered “good” and “useful,” respectively by participants. Individuals with higher gambling severity scores were more likely to perceive the tool as useful than those of the lower risk categories. Individuals with higher financial well-being were more likely to perceive the tool as useful and acceptable compared to those in lower financial well-being categories. Higher usability scores corresponded with higher tool acceptability. Previous efforts to monitor and track gambling spend was associated with acceptability and perceived usefulness. Gambling frequency and the number of gambling accounts held were not related to acceptability and perceived usefulness. Qualitative feedback included participant identified suggestions for improvements such as a need for interactive elements, a more detailed view of gambling expenditure, automation of activity statement upload functionality, more challenging and positively framed quiz content, and an emphasis on data security.

**Conclusions:**

The study provides preliminary support for the acceptability and perceived usefulness of a newly developed tool to increase awareness of gambling expenditure and knowledge on how gambling products function, providing directions for future improvements to the tool.

## Introduction

### Background

Gambling is a regulated and highly accessible activity worldwide. In Australia, 65% of adults gamble annually, while 46% of adults gamble annually globally [1,2]. Online betting, predominately race and sports betting, also referred to as wagering, has substantially altered betting participation. In 2024, 33% of Australian adults reported gambling online, a substantial increase from 8% in 2017, and 11% in 2021 [3]. Monthly sports bettors experience moderate to severe gambling harms at a higher rate (23%) than the broader population of regular gambling customers (8%) [4].

The experience of severe harms can contribute to developing gambling disorder, a behavioral addiction within the *DSM-5* (*Diagnostic and Statistical Manual of Mental Disorders, Fifth Edition*), which is characterized by persistent, recurrent maladaptive patterns of gambling behavior that is associated with significant distress and impairment [5]. Gambling disorder impacts quality of life and impairs functioning, with harms experienced across multiple life domains, including relationship difficulties, health problems, psychological distress, financial problems, work or study issues, and criminal activity [6]. These harms extend beyond the individual, affecting an estimated six to ten others for every person experiencing problem gambling [7]. Individuals may experience significant harms without a diagnosis. Australian research suggests that a larger proportion of regular gambling consumers experience low to moderate harms, compared to severe harms [1,8,9], highlighting that early intervention and prevention strategies are essential to reduce gambling harms.

Gambling harm-minimization policies and interventions have traditionally focused on interventions for individuals experiencing severe gambling-related harms. Public health campaigns typically highlight risks, low odds of winning, and indicators of gambling problems, placing primary responsibility on individuals to limit their gambling and recognize emerging harms [10]. Consumers generally view such public health messages as ineffective in changing behavior [11]. Despite growing international attention to the wide-reaching impacts of gambling harms, there remains little empirical evidence on how to best support consumers to gamble in a lower-risk manner and provide tools which meet their unique needs. This research directly addresses this gap by asking: Will regular betting customers likely use a personalized digital prevention tool designed to strengthen informed, sustainable decision-making about gambling?

### Digital Gambling Harm-Prevention Strategies

Regulators in most jurisdictions require licensed gambling operators to provide harm minimization and prevention tools such as deposit limits to all their customers, however, despite awareness for existing tools, customer uptake remains low [12-15]. A major barrier to tool use is the perception that these are only relevant for individuals with gambling problems [15-17]. Tools are often positioned under headings referring to ‘help’ or ‘gambling problems’ and refer to risk and treatment which may contribute to this perception. Other barriers include resistance to self-imposed limit setting and a desire to self-manage gambling without using external controls [16,18,19]. Despite low uptake, many customers hold positive attitudes towards harm minimization tools and report interest in using the tools to stay in control of their betting and avoid harms [12,16], highlighting a behavior-intention gap. Tools intentionally designed to meet consumer needs and framed in a positive and nonjudgemental manner may better contribute to gambling harm prevention efforts.

Gambling spend that exceeds personal affordability is a core behavior related to problematic gambling and an important target for harm reduction efforts. Financial impacts are among the most experienced negative consequences of gambling among those with and without gambling problems [20,21] and financial stress partially accounts for the relationship between problem gambling and psychological distress, depression, well-being, and gambling-related family impacts [22]. Online gambling has advantages over cash-based formats as it creates a digital record of transactions. Nonetheless, a clear statement of spend and net outcomes is typically unavailable, or only available on request, and these provide fragmented data due to the tendency for people to bet with numerous providers [23]. Consequentially, most regular gambling customers cannot accurately estimate their gambling spend [24,25]. In one Australian study, less than 5% of regular online bettors were able to estimate their past-month losses within a 10% margin of their actual outcome, with most underestimating their losses [26]. Those with greater losses were more inaccurate and were more likely to underestimate their losses. Individuals who spent more than they wanted to lose were more likely to have high problem gambling severity scores and spent and lost considerably more than those who adhered to the levels they were comfortable losing. These findings indicate that an enhanced awareness of spend would target an important group for harm reduction.

Recent policies by several international gambling regulators target affordability and enhanced awareness of spend in harm minimization policies. In the United Kingdom, online gambling operators are required to conduct affordability and financial risk checks based on specific spend metrics [27,28]. The Spanish gambling regulator is currently developing a dashboard where customers can view their spend across regulated online gambling operators [29]. In Australia, online wagering operators are required to send active customers a monthly statement of their spend, wins, losses, and net outcomes including a clear visual summary. An initial evaluation of this policy found 57% of participants read their statements “at least sometimes” and among those who did, 18% reported that the statement decreased their betting [23]. Frequent bettors and those with higher problem gambling severity scores reported greater subsequent reduction in their gambling. However, no behavioral changes were observed in an analysis of account transactions, suggesting that the spend summaries impact cognitions, attitudes, and intention rather than resulting in immediate behavioral change. This is consistent with evidence that information-based education is insufficient to change behavior, whereas tools that build real-time capability, reflection and applied skills are more effective in improving financial decision-making [30,31].

### Development of a Digital Tool to Enhance Gambling Expenditure Awareness to Inform Betting Decision-Making

This study aims to investigate acceptability and perceived usefulness of a novel digital tool designed to support informed and sustainable decision-making by increasing awareness of gambling expenditure. The tool, “BetWell,” is grounded in research, psychological theory, and behavioral science principles. BetWell was developed following extensive research to understand attitudes toward, engagement with, and impacts of existing harm minimization tools for gambling and ways to enhance acceptance and engagement [13,16,32-34]. BetWell uses positive, nonjudgmental language and avoids reference to terms such as “help,” “problems,” or “risk,” which may increase stigma and reduce perceived relevance [35]. The tool emphasizes that it is by “bettors, for bettors,” reflecting its development through extensive co-design and iterative feedback with regular betting customers. Developing interventions without input from the end-user can reduce subsequent attitudes, perceived usefulness and uptake [36], and fails to benefit from the relevant experience and knowledge of people with lived experience of the behavior that the intervention aims to target.

Our co-design procedure involved initial prototype design and development with clinicians (n=6), industry professionals (n=6), and regular gambling consumers (n=15), followed by participatory design workshops with regular gambling consumers (n=7), where mock ups of initial prototypes were displayed to gain feedback. Think aloud interviews were conducted with regular gambling consumers (n=11) after a minimum viable functional alpha prototype was developed; participants viewed the website and shared their perceptions. Throughout this process, we also gathered insights from an advisory board of consumers with lived experience of gambling harms (n=4). User feedback informed subsequent prototype iterations by removing items with low perceived usefulness (eg, initial mock ups contained an extensive goal setting feature using motivational interviewing techniques), expanding on features considered most valuable (eg, the gambling statement summary feature), and addressing potential limitations (eg, the prototypes were viewed as targeting individuals with problem gambling only, therefore we further edited the language to be more neutral and inviting for all regular gambling consumers). Our research with consumers and professionals revealed barriers to using existing harm minimization tools, which were consistent with previous studies [37,38], reflecting that restrictive tools were seen as irrelevant, burdensome to use, and offer limited immediate perceived benefits. Regular bettors stated that they would be more likely to use a tool that offers clear, immediate benefits, and emphasized positive outcomes rather than reducing negative outcomes. These findings influenced the subsequent design of the alpha prototype.

The BetWell tool examined in this study incorporates behavioral change technique active ingredients by Michie et al [39]. A key function of BetWell enables users to view a spend summary across all the gambling sites they use, including their total spend, wins, losses, and net outcomes, while also presenting a graphic summary. The tool displays personalized feedback about their past month gambling spend including providing future (eg, yearly) spend projections based on past spend, what their spend represents in terms of the percentage of their income and hours of work, as well as alternate spend options in comparison to necessary and discretionary options (eg, groceries and holidays). These features aim to assist users to actively monitor and reflect on their spending within their personal context and reflect on the extent that it aligns with their goals. As such, BetWell’s features aligns with the Feedback and Monitoring, and Goals and Planning behavioral change techniques [39]. BetWell also addresses common cognitive biases and offers psychoeducational feedback on how betting outcomes are determined and evidence-based behavioral strategies for safer betting behaviors via an interactive quiz. This feature aligns with Shaping Knowledge, Natural Consequences, Identity (Reframing), and Comparison of Behavior based on the content BetWell provides.

Acceptability studies play a key role in intervention development and evaluation and serve as a necessary precursor to implementing digital mental health interventions [40,41]. Low uptake, even when tools are effective, remains a common challenge faced in digital mental health [42]. Without establishing acceptability, uptake and engagement may continue to be low regardless of the intervention’s effectiveness [36]. Within the Technology Acceptance Model (TAM), perceived usefulness and ease of use are key constructs predicting intention to use and actual adoption of digital interventions [43].

### Objectives

This exploratory study aimed to investigate acceptability and perceived usefulness of the novel digital tool, BetWell, framed as a positive, user-centered resource to enable regular bettors to enhance their gambling experience by viewing a personalized data tracking dashboard. This study investigated three exploratory research questions (RQs) to gain overall acceptability and additional feedback on the digital tool among Australian online bettors: (RQ1) how do participants perceive BetWell and what refinements do they recommend? (RQ2) How does acceptability and usefulness vary based on perceived usability, problem gambling severity, financial well-being, number of gambling accounts used, gambling frequency, tracking of gambling spend, and use of activity statements? (RQ3) Which factors independently predict acceptability and perceived usefulness when accounting for life satisfaction, gambling satisfaction, and demographics?

## Methods

### Overview

The study used a mixed methods cross-sectional design using a convergent approach, in which quantitative survey measures and qualitative open-ended responses were collected concurrently via an online survey to assess acceptability and perceived usefulness of BetWell. The study was approved by the University of Sydney’s Human Research Ethics Committee (protocol number 2024/HE000747). Reporting is aligned with the Checklist for Reporting Results of Internet E-Surveys (CHERRIES) [44].

The study was preregistered on the Open Science Framework [45]. Some deviations from the preregistered plan occurred. The originally proposed hypotheses were removed because the existing literature did not provide sufficient empirical evidence to justify directional predictions. These were replaced with exploratory research questions, which remained conceptually consistent with those specified in the preregistration but were revised slightly to reduce overlap in wording.

### Participants

Participants were recruited via an Australian market research company in May 2025. Participants were eligible to participate in the study if they met the following criteria: (1) were over 18 years old, (2) placed bets with at least two major Australian online wagering operators in the previous month and had access to their gambling activity statements provided by a gambling operator (activity statement) to be uploaded to the tool, (3) could speak, read, and write English, and (4) were currently not in formal treatment related to their gambling. Those who expressed interest were directed to the participant information sheet and informed of the consent procedures, followed by the eligibility screening questions. Participants were linked to the digital tool with instructions for use and an outline of tasks to complete before being redirected to an online survey delivered on the secure survey platform, Qualtrics. Participants were reimbursed with credit valued at 20 AUD (1 AUD=US $0.69 conversion). All participants were provided with contact information about available 24-hour support services if they experienced any distress or needed help for problems related to gambling.

A power analysis conducted using G*Power [46] indicated that a sample size of 111 participants was required to detect a small effect (power 0.95 and alpha 0.05). Based on initial screening on the market research platform, 27,003 individuals were initially invited to participate in the study and 12,854 were sent reminder invitations. The survey yielded 213 complete responses, however, responses were removed if they failed attention checks (n=23), were duplicate responses (n=33), or had an implausible response duration (n=17). The final sample included responses from 140 participants. The final dataset contained no missing quantitative data as participants were required to complete all survey items to progress. However, the qualitative feedback was optional, and not all participants provided responses.

### Procedure

Following eligibility confirmation, participants accessed a working version of BetWell via a private link in the Qualtrics survey and completed simple instructions to test the website’s functionality, including creating an account, estimating their past month spend and how much they were comfortable losing in the subsequent month, uploading their own activity statements, viewing their personalized dashboard and engaging with the quiz. Participants were required to engage with the tool for a minimum of 10 minutes during which the survey was locked, before returning to complete the survey.

### The BetWell Tool

The BetWell tool consisted of the following items:

1. Pre-data check-in: Before viewing their data, users are prompted to estimate their net result for the previous month and set an intention for how much they are comfortable losing in the following month. This structured reflection point surfaces the gap between perceived and actual outcomes before the user views their data, making the subsequent information more meaningful and personally relevant.
2. Aggregated spend dashboard: BetWell consolidates betting activity across all operators into a single longitudinal view via the “statement summary” feature. Individuals can see cumulative losses over time alongside net outcomes, visualized monthly, biannually, yearly, or across the lifetime. This directly addresses the documented inability of bettors to accurately recall their expenditure by shifting cognition from episodic recall to pattern recognition.
3. Alternative spend comparison: Gambling expenditure is translated into everyday consumption equivalents, familiar, concrete, and personally relevant via the “Spend Strategy” feature. This converts abstract monetary loss into tangible trade-offs without moral framing, drawing on established decision science principles around opportunity cost and concreteness effects in valuation.
4. Psychoeducational quiz: An interactive “quiz” feature building understanding of how betting products work and common cognitive biases. The multiple-choice quiz prompts users to reflect on common betting scenarios and how they would respond. Responses provide nonjudgmental feedback focused on enhancing sustainable gambling. Refer to Multimedia Appendix 1 for screengrabs of BetWell’s features.

### Measures

Acceptability of BetWell was assessed using a 5-point Likert scale. The original Adapted-Mobile Application Rating Scale (A-MARS) had high internal consistency (Cronbach α=.721-.920) [47]. The subscale items assessed in our study included engagement, interest, ease of use, navigation, layout, quality of quiz information, whether they would recommend the tool, and tool subjective quality ratings. These A-MARS items demonstrated good internal consistency in the current sample (Cronbach α=.888). The 5-point subscale items are interpreted as 1-inadequate, 2-poor, 3-acceptable, 4-good, and 5-excellent [48]. Total scores range from 8 to 40 with higher scores indicating higher acceptability.

Perceived usefulness of BetWell was assessed using five author-generated questions pertaining to the specific features within BetWell, measured using a five-point Likert scale. The five items were variates of the following: “please indicate how useful this (eg, activity statement summary feature) was.” These five items showed good internal consistency (Cronbach α=.882). Each question was presented alongside an image, using the screenshots presented in Multimedia Appendix 1, to remind participants of the specific feature being referenced. Response options ranged from 1 “not at all useful” to 5 “very useful” for all questions based on the format used by digital health studies to assess perceived usefulness [49,50]. Total scores ranged from 5 to 25, with higher scores indicating higher perceived usefulness.

Perceived usability of BetWell was assessed using a 10-item measure, the System Usability Scale (SUS) [51]. This scale demonstrates strong internal consistency and concurrent validity across systems and contexts [51,52]. Scores range from 0 to 100, with higher scores indicating higher usability. Scores are interpreted as worst imaginable-12.5, awful-20.3, poor-35.7, ok-50.9, good-71.4, excellent-85.5, and best imaginable-90.9 [53].

Problem gambling severity was measured using the Problem Gambling Severity Index (PGSI) [54], a validated 9-item screen utilizing four response options “never,” “sometimes,” “most of the time,” and “almost always” which has good internal consistency (Cronbach *α*=.86) [55]. Survey items were summed to provide a total score ranging from 0‐27. Scores were categorized as 0 “No Risk,” 1‐3 “Low Risk,” 4‐7 “Moderate Risk” and scores >8 “Problem Gambling.”

Financial well-being was assessed using a five-item Commonwealth Bank of Australia and the Melbourne Institute Financial Wellbeing Scale (R-5) [56]. The scale was developed and validated in Australia with the R-5 demonstrating good internal consistency (Cronbach α=.86) [56]. Total response value scores were calculated and multiplied by five. Higher scores indicate higher financial well-being, and total scores were categorized into four groups: “Having Trouble,” “Just Coping,” “Getting By”, and “Doing Great.

Gambling frequency was assessed using author-generated questions asking frequency of gambling across formats (ie, pokies, casino table games, poker, lotteries, scratch tickets, race wagering, sports wagering, keno or bingo, private betting, and other). Responses options included “never,” “less than once per month,” “1-3 time per month,” “1-3 times per week,” and “4+ times per week.” Participants’ highest reported gambling frequency across activities was used as their overall gambling frequency.

Activity statement use was assessed using an author generated item. Respondents were asked “please indicate how often you have opened and read a monthly activity statement” with response options ranging from 1 “Never” to 5 “Always.”

Gambling spend tracking was assessed using an author-generated item. Respondents were asked “do you track how much money you spend on gambling?” with response options range from 1 ‘Never” to 5 “Always.”

The number of gambling accounts held by participants was assessed using an author-generated item asking, “how many online gambling accounts have you used in the past 12-months?” with response options on a 5-point scale. Response options were “1,” “2,” “3,” “4,” and “5+” corresponding to the number of accounts.

Life Satisfaction was assessed by asking participants to rank their life satisfaction on a scale of 0 to 10 (from totally dissatisfied to totally satisfied) [57]. Scores were allocated into four groups: “Very Satisfied” (a score of 9 or 10), “Satisfied” (a score of 7 to 8), “Not so Satisfied” (a score of 4 to 6) and “Dissatisfied” (a score of 0 to 3) [57].

Gambling Satisfaction was assessed by asking participants to rank their gambling satisfaction on a scale of 0 to 10 (from totally dissatisfied to totally satisfied) [58]. Scores were allocated into four groups: “Very Satisfied” (a score of 9 or 10), “Satisfied” (a score of 7 to 8), “Not so Satisfied” (a score of 4 to 6) and “Dissatisfied” (a score of 0 to 3).

Participant demographic information was assessed via six items regarding their age, sex, income, employment, language, and education.

Qualitative feedback on the tool was assessed via four author generated questions following the perceived usefulness items. Participants could choose to respond to the following questions: (1) “How would you improve this Activity Statement Summary feature?,” (2) “How would you improve this Quiz feature?,” (3) “How would you improve this Spend Strategy feature?,” and (4) “Do you have any additional feedback on the tool?”

### Data Analysis

Quantitative survey data was analyzed using jamovi (version 2.6.44; The jamovi Project) and R (version 4.5.1; R Core Team). Descriptives statistics were conducted to obtain means, SDs, and proportions. Regression models were conducted to identify associations between variables. Where linear regression model assumption testing for multicollinearity (variance inflation factor less than 5 was deemed acceptable), normality (Shapiro-Wilk test *P*>.05 was deemed acceptable), linearity (residual plots showing a linear relationship was deemed acceptable), independence of errors (Durbin-Watson statistic scores between 1.5 and 2.5 were deemed acceptable), or homoscedasticity (Breusch-Pagan test *P*>.05 is deemed acceptable) was violated, robust linear regressions were conducted. Where residuals deviated from normality and where the heteroscedastic assumption was violated, bootstrapped CIs (5000 resamples) and heteroskedasticity-consistent SEs were computed to ensure robust inference. Where exploratory models were conducted with a large number of predictors, to identify the most important predictors while accounting for multicollinearity, a Least Absolute Shrinkage and Selection Operator (LASSO) regression was conducted using the *glmnet* package in R [59-61]. Predictors retained by the LASSO were subsequently entered into a multiple linear regression model to estimate regression coefficients and statistical significance.

The variables assessing acceptability, perceived usefulness, perceived usability, and age were tested as continuous variables, while gambling spend tracking and activity statement use were tested as ordinal variables. Financial well-being, gambling severity, gambling frequency, life satisfaction, gambling satisfaction, sex, language, income, employment status, and education were tested as categorical variables. All models were robust linear regressions due to normality assumption violations.

Qualitative data derived from open-ended survey feedback was analyzed using inductive conceptual content analysis to identify insights into the perceived acceptability, usefulness, and usability of the tool and identify content and design suggestions for tool improvements. The qualitative feedback was considered complementary to the quantitative components, allowing participants to provide further details on their acceptability, perceived usefulness, and usability ratings. The coding framework was reviewed by two authors (DC and SG) and data coding was completed by one author (DC). Where segments of the feedback responses overlapped across more than one coded category, the coder selected multiple categories.

## Results

### Sample Characteristics

The sample comprised of 140 participants, who were predominately English-speaking (n=125, 89%), males (n=110, 75%), in full-time employment (n=114, 81%), with tertiary education (n=88, 63%), household income at or greater than US $71,760 (n=72, 51%), with a mean age of 41.3 (SD 10.9) years.

More than two-fifths (60/140, 42%) of participants were classified as having no or low risk of gambling problems, one-third (46/140, 32.9%) were at moderate risk, and one-quarter (34/140, 24.7%) were classified as having a severe gambling problem. Most participants (80/140, 57.1%) were categorized as “getting by” financially, although 15% (21/140) had lower financial well-being (struggling or just coping). Most of the participants (110/140, 79%) were at least “satisfied” with their life and 65% (91/140) were at least “satisfied” with their gambling.

Most participants (124/140, 89%) gambled at least weekly and most frequently held 2 gambling accounts (43/140, 31%). A total of 77% (108/140) of participants reported tracking their gambling spend at least “sometimes,” while 71% (99/140) reported reading their monthly activity statements at least “sometimes.” Refer to Table 1 for a full list of participant characteristics.

**Table 1.** Participant characteristics (N=140).

	Summary, n (%)
Age, mean (SD)	41.3 (10.9)
Sex	
Male	110 (75.3)
Female	35 (24)
Prefer not to answer	1 (0.7)
Language other than English spoken at Home	
No	125 (89.3)
Yes	15 (10.7)
Education	
Secondary or high school education	21 (15)
Post secondary education (certificate III, certificate IV, or advanced diploma)	31 (22.1)
University degree (bachelors)	64 (45.7)
Graduate diploma or graduate certificate	8 (5.7)
Postgraduate degree (master’s degree, doctorate)	16 (11.4)
Employment	
Employed full time	114 (81.4)
Employed part time or casual	19 (13.6)
Not currently employed	3 (2.1)
Retired	1 (0.7)
Full time student	1 (0.7)
Principally engaged in domestic duties	2 (1.4)
Annual household income (AUD)^a^	
$7800-$25,599	4 (2.8)
$26,000-$51,999	12 (8.5)
$52,000-$77,999	25 (17.9)
$78,000-$103,999	23 (16.4)
$104,000-$155,999	28 (20)
$156,000 or more	44 (31.4)
Not stated	4 (2.9)
Problem Gambling Severity Index	
No risk	25 (17.1)
Low risk	35 (25.3)
Moderate risk	46 (32.9)
Problem gambling	34 (24.7)
Financial well-being	
Having trouble	6 (4.3)
Just coping	15 (10.7)
Getting by	80 (57.1)
Doing great	39 (27.9)
Gambling frequency	
1‐3 times per month	16 (11.4)
1‐3 times per week	88 (62.9)
4+ times per week	36 (25.7)
Gambling activity statement use	
Never	9 (6.4)
Rarely	32 (22.9)
Sometimes	62 (44.3)
Very often	26 (18.6)
Always	11 (7.9)
Gambling spend tracking	
Never	9 (6.4)
Rarely	23 (16.4)
Sometimes	72 (51.4)
Very often	27 (19.3)
Always	9 (6.4)
Number of online gambling accounts	
1 account	17 (12.1)
2 accounts	43 (30.7)
3 accounts	33 (23.6)
4 accounts	32 (22.9)
5+ accounts	15 (10.7)
Life satisfaction	
Very satisfied	28 (20)
Satisfied	82 (58.6)
Not so satisfied	24 (17.1)
Dissatisfied	6 (4.3)
Gambling satisfaction	
Very satisfied	22 (15.7)
Satisfied	69 (49.3)
Not so satisfied	42 (30.0)
Dissatisfied	7 (5.0)

a Based on 1 AUD=US $0.69 conversion.

### Acceptability

**Figure 1.**
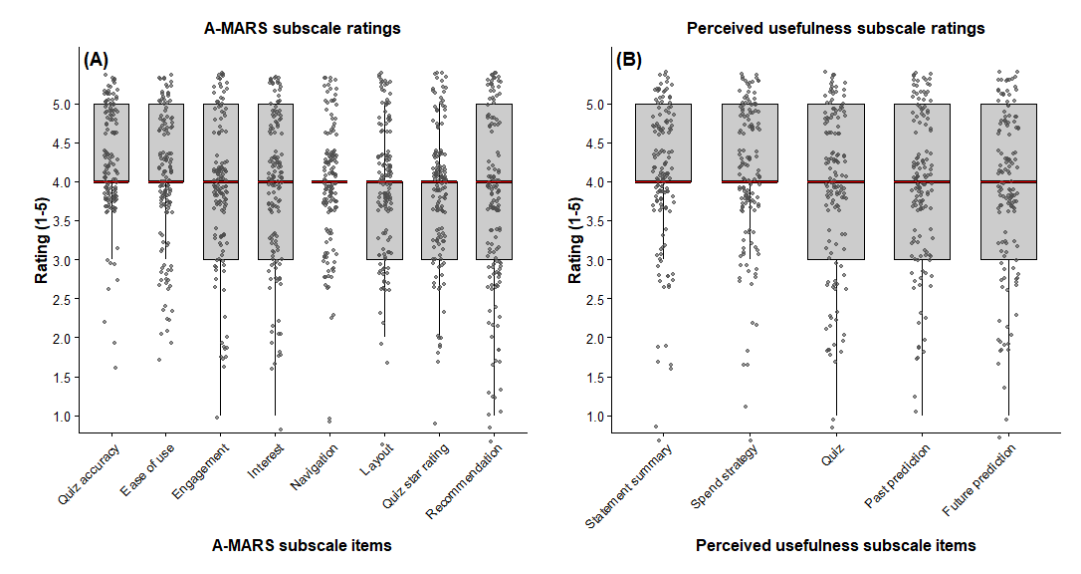
. (**A**) A-MARS (Adapted-Mobile Application Rating Scale), and (**B**) perceived usefulness subscale score distribution. Note. The box represents the interquartile range, the dots represent the individual data points (jitter), and the red lines represent the median. A-MARS: Adapted-Mobile Application Rating Scale.

The mean total A-MARS acceptability score was 32/40 (SD 5.20, range 12‐40). A-MARS mean scores for each subscale item were the following: quiz accuracy mean 4.3 (SD 0.66), easy to use mean 4.04 (SD 0.85), engaging mean 3.9 (SD 0.89), interesting mean 3.9 (SD 0.95), clear navigation mean 3.9 (SD 0.77), layout mean 3.9 (SD 0.80), quiz star rating mean 3.8 (SD 0.83), and recommendation mean 3.7 (SD 1.13). All scores fell within the ‘acceptable’ to ‘good’ cutoff ranges [48]. Figure 1A depicts the distribution of the subscale scores.

### Perceived Usefulness

The overall perceived usefulness score across participants was 20/25 (SD 3.9, range 5‐25; derived across the five main features of the tool). The subscale mean scores were the following: activity statement summary mean 4.1 (SD 0.87), spend strategy mean 4.1 (SD 0.9), quiz mean 3.9 (SD 1), past prediction mean 3.9 (SD 0.93), and future prediction mean 3.9 (SD 1). Figure 1B depicts the distribution of the subscale scores.

### Usability

The mean total perceived usability score on the SUS was 69/100 (SD 17.3, range 2.5‐100), exceeding the established ‘ok’ cut off criteria for usability [53]. The median score was 70 (IQR 22.5), and the mode was 73, indicating that most participants reported usability in the “ok” to “good” range.

### Statistical Analysis: Predictors of Acceptability and Usefulness

#### Usability

**Figure 2.**
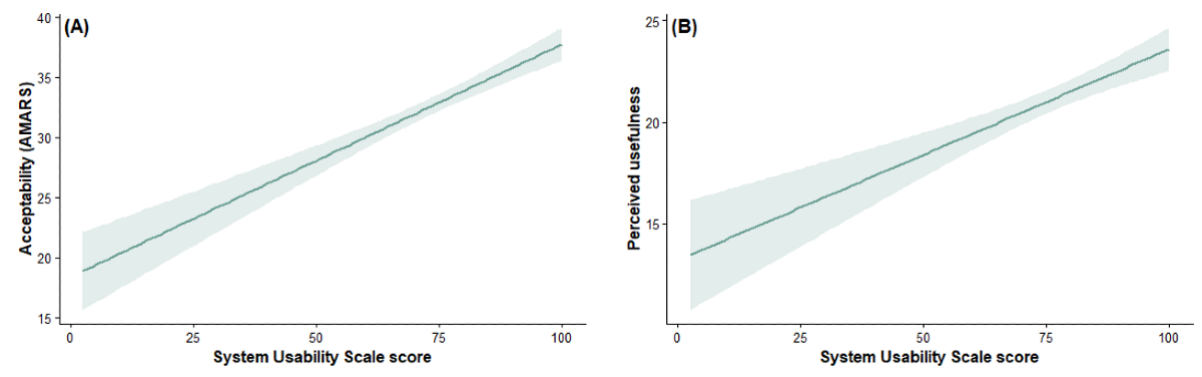
Associations between (**A**) perceived usability and acceptability, and (**B**) perceived usability and perceived usefulness. Note. The shaded area represents the 95% CI. A-MARS: Adapted-Mobile Application Rating Scale.

Usability was a predictor of acceptability (*R*^*2*^=.384, adjusted *R*^*2*^=.379, *F*_1,138_=86, and *P<*.001) and perceived usefulness (*R*^*2*^=.245, adjusted *R*^*2*^=.24, *F*_1,138_=44.8, and *P<*.001). Higher usability scores were associated with higher acceptability (*b*=0.186, SE=0.020, 95% CI 0.147-0.226, *β*=.62, *t*_138_ =9.27, and *P*<.001; Figure 2A) and higher perceived usefulness (*b*=0.112, SE=0.017, 95% CI 0.079-0.145, *β*=.50, *t*_138_ =6.69, and *P*<.001; Figure 2B).

#### Problem Gambling Severity

**Figure 3.**
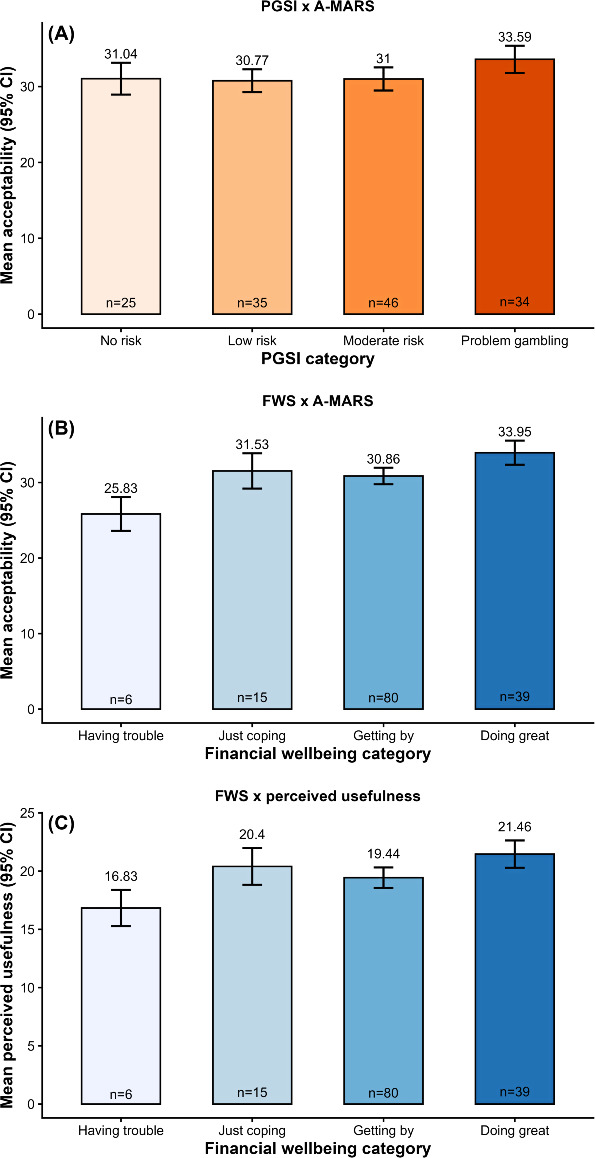
Subgroup differences in acceptability and perceived usefulness by gambling severity and financial well-being*.* Note. (**A**) Gambling severity and acceptability; (**B**) financial well-being and acceptability; and (C) financial well-being and perceived usefulness. Error bars represent confidence intervals and group mean values are presented above each corresponding bar. AMARS: Adapted-Mobile Application Rating Scale; FWS: Financial Wellbeing Scale; PGSI: Problem Gambling Severity Index.

PGSI was not a significant predictor of A-MARS acceptability (*R*^2^=.049, adjusted *R*^2^=.028, *F*_3,136_=2.31, and *P=*.08). PGSI was a significant predictor of perceived usefulness (*R*^2^=.096, adjusted *R*^2^=.076, *F*_3,136_=4.82, and *P=*.003). Post hoc comparisons with Bonferroni corrections revealed participants in the High Problem Gambling category perceived the tool as more useful compared to the No Risk (mean difference=2.88, SE=1.07, *t*_136_=2.69, *P*=.049), Low Risk (mean difference=2.69, SE=0.79, *t*_136_=3.43, and *P*=.005), and Moderate Risk (mean difference=2.86, SE=0.81, *t*_136_=3.54, and *P*=.003) categories (Figure 3A).

#### Financial Well-being

Financial well-being was a predictor of A-MARS acceptability (*R*^2^=.122, adjusted *R*^2^=.102, *F*_3,136_=6.29, *P<*.001). Post hoc comparisons with Bonferroni corrections revealed that the Having Trouble’ financially group showed lower acceptability compared to the “Just Coping” (mean difference =−5.70, SE (1.75), *t*_136_ =−3.25, *P*=.009), “Getting By” (mean difference =−5.03, SE=1.37, t_136_ =−3.68, *P*=.002), and “Doing Great” (mean difference =−8.12, SE=1.50, t_136_ =−5.43, *P*<.001) financially groups. The “Doing Great” financially participants also showed higher acceptability compared to the “Getting By” (mean difference =−3.09, SE=1.00, t_136_ =−3.09, *P*=.02) group. No other pairwise comparisons between financial well-being groups reached statistical significance (Figure 3B).

Financial well-being predicted BetWell perceived usefulness (*R*^2^=.081, adjusted *R*^2^=.061, *F*_3,136_=3.99, *P=*.009). Post hoc comparisons with Bonferroni corrections revealed that the “Doing Great” financially participants reported higher BetWell perceived usefulness compared to the “Getting By” (mean difference=2.02, *t*_136_=−2.68*, P*=.049) and “Having Trouble” financially participants (mean difference=4.63, *t*_136_ =−4.37, *P*<.001). In addition, the “Just Coping” financially participant reported higher perceived usefulness than those “Having Trouble” financially (mean difference=3.57, *t*_136_=−2.97, *P*=.02). No other pairwise comparisons between financial well-being groups reached statistical significance (Figure 3C).

#### Gambling Frequency

Gambling frequency did not predict acceptability (*R*^2^=.000, adjusted *R*^2^=.000, *F*_2,137_=0.03, *P=*.97) or perceived usefulness (*R*^2^=.019, adjusted *R*^2^=.005, *F*_2,137_=1.33, *P=*.27).

#### Activity Statement Use

**Figure 4.**
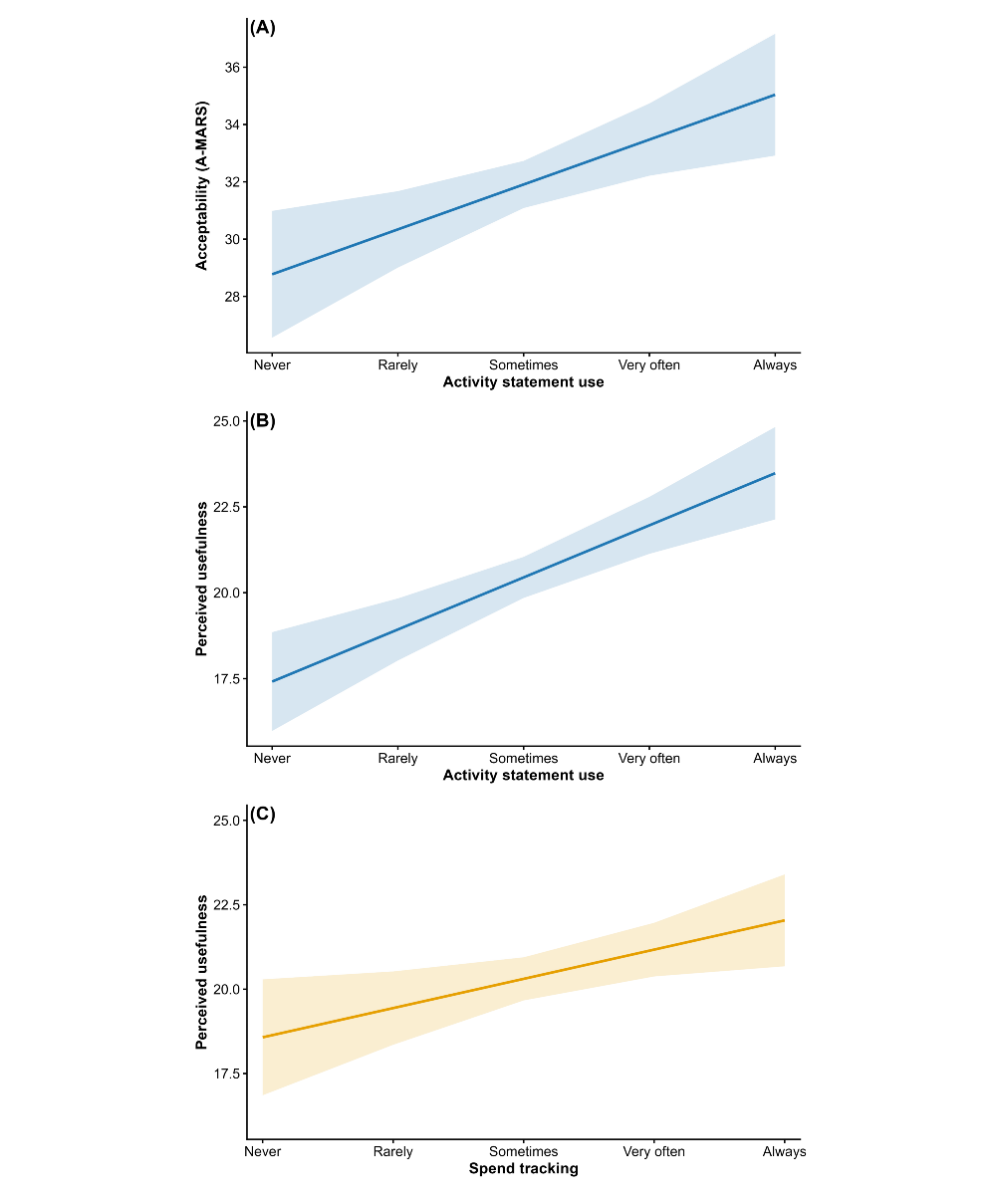
The association between (**A**) acceptability and activity statement use, (**B**) perceived usefulness and activity statement use, and (**C**) spend tracking and perceived usefulness*.* The shaded area represents the 95% CI. A-MARS: Adapted-Mobile Application Rating Scale.

Activity statement use was a predictor of acceptability (*R*^2^=.094, adjusted *R*^2^=.088, *F*_1,138_=14.4, *P<*.001) and perceived usefulness (*R*^2^=.180, adjusted *R*^2^=.174, *F*_1,138_=30.2, *P<*.001) with greater activity statement use corresponding with higher acceptability (*b*=1.60, *SE*=0.53, 95% CI 0.64‐2.71, *β*=.31, *t*_138_ =3.00, *P*=.003; Figure 4A) and higher perceived usefulness (*b*=1.66, *SE*=0.37, 95% CI 0.96‐2.44, *β*=.42, *t*_138_ =4.46, *P*<.001; Figure 4B).

#### Gambling Spend Tracking

Gambling spend tracking did not predict acceptability (*R*^2^=.023, adjusted *R*^2^=.016, *F*_1,138_=3.31, *P=*.07) but was a predictor of perceived usefulness (*R*^2^=.051, adjusted *R*^2^=.044, *F*_1,138_=7.46, *P=*.007) with higher gambling tracking frequency corresponding with higher perceived usefulness (*b*=0.94, *SE*=0.36, 95% CI 0.27‐1.69, *β*=.23, *t*_138_=2.61, *P*=.01; Figure 4C).

#### Number of Gambling Accounts

The number of accounts held did not predict acceptability (*R*^2^=.052, adjusted *R*^2^=.024, *F*_4,135_=1.87, *P=*.12) or perceived usefulness (*R*^2^=.066, adjusted *R*^2^=.039, *F*_4,135_=2.40, *P*=.05).

#### Predictors of Acceptability

A LASSO regression with cross-validation was conducted to identify key predictors of acceptability. The following variables were entered into the LASSO regression model: usability, gambling severity, financial well-being, gambling frequency, activity statement use, spend tracking, number of accounts, life satisfaction, gambling satisfaction, and demographic variables (age, income, education, language, sex, and employment. Four demographic variables (income, education, language, and sex) were shrunk to zero and excluded. The remaining predictors were included in a multiple linear regression. The overall model was significant (*R*^2^=.67, adjusted *R*^2^=.491, *F*_16,123_=3.73, and *P*<.001). Usability was a significant predictor in the model. PGSI comparisons between the No Risk category and the Moderate Risk and Problem Gambling categories reached significance in the model (Table 2).

**Table 2.** Exploratory predictor model of acceptability.

Predictor	Estimate	SE	Lower, 95% CI	Upper, 95% CI	Two-tailed *t* test *(df)*	*P* value
Intercept	11.46	3.12	5.27	17.65	3.66 (123)	<.001
Usability	0.20	0.02	0.16	0.24	10.20 (123)	<.001
Age	−0.020	0.0307	−0.0802	0.04	−0.63 (123)	.53
PGSI:						
Low risk – no risk	1.16	1.01	−0.83	3.15	1.16 (123)	.25
Moderate risk – no risk	2.00	0.98	0.06	3.93	2.05 (123)	.04
Problem gambling – no risk	5.12	1.13	2.87	7.37	4.51 (123)	<.001
Gambling frequency:						
1‐3 times per week – 1‐3 times per month	−0.29	1.05	−2.36	1.79	−0.28 (123)	.78
4+ times per week – 1‐3 times per month	−1.81	1.21	−4.20	0.58	−1.50 (123)	.14
Financial well-being:						
Just coping – having trouble	0.78	3.00	−5.17	6.72	0.26 (123)	.80
Getting by – having trouble	0.55	2.80	−4.98	6.09	0.20 (123)	.84
Doing great – having trouble	0.06	2.88	−5.65	5.77	0.02 (123)	.98
Life satisfaction:						
Not so satisfied – dissatisfied	4.64	2.96	−1.21	10.50	1.57 (123)	.12
Satisfied – dissatisfied	6.00	3.10	−0.14	12.14	1.93 (123)	.06
Very satisfied – dissatisfied	5.72	3.21	−0.63	12.08	1.78 (123)	.08
Gambling satisfaction:						
Not so satisfied – dissatisfied	−0.88	1.72	−4.28	2.52	−0.51 (123)	.61
Satisfied – dissatisfied	−0.07	1.85	−3.73	3.59	−0.04 (123)	.97
Very satisfied – dissatisfied	1.22	1.98	−2.70	5.14	0.62 (123)	.54

#### Predictors of Perceived Usefulness

A LASSO regression with cross-validation was conducted to identify key predictors of perceived usefulness. The following variables were entered into the LASSO regression model: usability, gambling severity, financial well-being, gambling frequency, activity statement use, spend tracking, number of accounts, life satisfaction, gambling satisfaction, and demographic variables (age, income, education, language, sex, and employment). PGSI, gambling frequency, number of accounts, gambling satisfaction, and six demographic variables (age, income, education, language, sex, and employment) were shrunk to zero and excluded. The remaining predictors (usability, financial well-being, spend tracking, activity statement use, and life satisfaction) were retained in the multiple linear regression. The overall model was significant (*R*^2^=.442, adjusted *R*^2^=.375, *F*_15,124_=6.55, *P*<.001). Usability was a significant predictor in the model. Activity statement use comparisons between the “Never” having opened and read activity statements and those who “Sometimes” or “Very Often” used activity statements reached statistical significance (Table 3).

**Table 3.** Exploratory predictor models of perceived usefulness.

Predictor	Estimate	SE	Lower, 95% CI	Upper, 95% CI	Two-tailed *t* test *(df)*	*P* value
Intercept	10.45	1.95	6.58	14.31	5.35 (124)	<.001
Usability	0.08	0.02	0.05	0.12	4.96 (124)	<.001
Financial well-being:						
Just coping – having trouble	4.36	2.48	−0.55	9.27	1.76 (124)	.08
Getting by – having trouble	2.67	2.35	−1.98	7.32	1.14 (124)	.26
Doing great – having trouble	3.99	2.45	−0.86	8.83	1.63 (124)	.11
Life satisfaction:						
Not so satisfied – dissatisfied	−1.53	2.39	−6.24	3.187	−0.64 (124)	.52
Satisfied – dissatisfied	−1.22	2.39	−5.94	3.51	−0.51 (124)	.61
Very satisfied – dissatisfied	−2.11	2.47	−7.00	2.77	−0.86 (124)	.39
Track spend:						
Rarely – never	−2.07	1.37	−4.79	0.65	−1.51 (124)	.13
Sometimes – never	−0.66	1.28	−3.19	1.87	−0.52 (124)	.61
Very often – never	−0.90	1.38	−3.63	1.83	−0.65 (124)	.51
Always – never	−1.02	1.71	−4.40	2.36	−0.60 (124)	.55
Activity statement use:						
Rarely – never	1.25	1.32	−1.35	3.86	0.95 (124)	.34
Sometimes – never	3.29	1.26	0.80	5.79	2.61 (124)	.01
Very often – never	5.16	1.40	2.39	7.93	3.68 (124)	<.001
Always – never	3.17	1.65	−0.09	6.43	1.92 (124)	.06

### Qualitative Feedback

More than 80% of participants provided feedback on the three features of BetWell (123/140, 88% provided feedback on the activity statement feature, 122/140, 87% for the quiz, and 116/140, 83% on the spend strategy), and 49% (69/140) provided feedback on the overall tool. The qualitative feedback served as an optional opportunity for participants to provide additional feedback on their perceptions towards the tool to complement the qualitative findings. This feedback was categorized based on whether it related to the tool’s acceptability, perceived usefulness, or usability, and these findings are displayed in Table 4 using the joint displays approach [62].

**Table 4.** Overview of participant feedback corresponding with tool acceptability, usefulness, and usability.

Theme and description of findings	Illustrative quotes
Acceptability	
Information could be presented in a more engaging format.	“*Use green highlights or badges to show when a user improves, such as spending less or reducing losses, to encourage safer habits.”* *“Incorporate more interactive elements, provide actionable tips, and offer customizable reporting.”* *“I think the red and black colors are a bit too harsh”*
The statement summary feature is considered acceptable by some participants and does not require any changes.	*“Best thing I have seen.”* *“Doesn’t need improving.”*
The spend strategy comparisons could be more relevant to users.	*“It could provide more relevant information on spending estimates.”*
The spend strategy content is informative.	*“It gives a really good indication of your spending in comparison to earnings.”* *“I think this was good, it showed where my money was going and what it could be.”*
The quiz content is negatively focused.	*“It’s all based on losing. How about when you win?”*
Considerations for information security and privacy.	*“Give user the confidence [that] private information is protected.”*
Participants showed openness to increase their level of engagement with the tool.	*“Incorporate goal-setting tools and real-time spending alerts.”*
Usefulness	
More detailed analysis across specific time periods to identify where money is spent.	*“Let users switch between monthly or weekly breakdowns to better track gambling patterns and changes over time.”*
Feedback may be used to look at betting patterns with the aim of improving their strategy rather than increase awareness.	*“It needs more detail on where you lost money, like what sport or team.”*
Feedback suggested the lack of novelty of the statement summary information and a lack of relevance to participants.	*“I already have this info on statement.”* *“It is quite simplistic and the sort of thing most people could track in their head.”* *“I don’t think it needs improving, I just wouldn’t use it”*
The spend strategy is considered useful in contextualizing spend.	*“[I] found it a useful illustrative example”* *“It shows what you could be spending your money on which would be more beneficial. Rams home the impact of losing money on gambling.”* *“I actually think that the spend strategy is fantastic. I think people might be surprised by some of those options. Like how much they spend a year, what it could equate to in regards to petrol. So I wouldn’t change a thing, I think that’s actually a great feature.”*
The spend strategy may not be relevant for all participants.	*“It is straight forward but just not really useful for most average gamblers.”* *“It seems like a false equivalence putting it against other household features.”*
Mixed responses were provided on the usefulness of the quiz content including its perceived lack of novelty.	*“I thought the quiz was great, it was useful and also helped me understand gambling.”* *“Quite informative but not sure how useful, needs work.”* *“The questions were very generic and don’t say anything the addict doesn’t know”* *“I’m not sure if the quiz is needed”* *“I’m aware of this already”*
The quiz content could be more challenging.	*“Maybe creating a hard quiz for people who are kind of aware of the pitfalls of gambling.”* *“The quiz could have more challenging questions.”*
The quiz is considered useful for only one use.	*“It is a bit of fun, but once you’ve done it, why would you do it again.”* *“It’s a one and done feature.”*
Usability	
Text could be clearer to read and understand.	*“It needs to be simple and clear with personalisation.”* *“Maybe make the statement summary boxes a bit clearer in terms of what periods they indicate.”*
Improving the statement uploading functionality was discussed including reducing friction by automating statement uploads and allowing for multiple simultaneous statement uploads.	*“Maybe if it were able to be imported directly from betting platform”* *“I would make it so you could upload multiple statement at once.”* *“The statements tend to not load or crash. Took a few attempts to finally upload the statements and for it to reflect back.”*
The website’s ease of navigation can be improved.	*“Was a little hard to navigate to begin with.”*

## Discussion

### Principal Findings

BetWell was considered acceptable and useful based on both descriptive statistics and qualitative feedback from a sample of regular gambling customers. Responses varied, which is expected given participant heterogeneity and the evaluation of a tool in its prototype stage. Most participants also rated BetWell as usable, which is promising given that the tool prototype was designed to be functional, but not ready for implementation. The prototype required participants to manually download activity statements from their email and upload them onto BetWell. This is a friction point that will be removed in future iterations by allowing for automatic statement transfers into BetWell, which is expected to enhance usability scores, in accordance with the Technology Acceptance Model [43].

Perceived usefulness of BetWell varied between users based on problem gambling severity. Individuals with higher problem gambling severity rated the tool as more useful, providing initial support for BetWell’s value among those already experiencing gambling related harms. Problem gambling severity also independently predicted usefulness, demonstrating the strength of this relationship. This finding aligns with studies showing that individuals experiencing gambling problems are more likely to use behavioral strategies [63,64] and perceive value in viewing their activity statements than those with lower reported gambling problems [15,23]. Importantly, individuals with lower problem gambling severity also rated BetWell as useful, based on a mean perceived usefulness score of 20 (out of 25), suggesting BetWell’s perceived relevance for harm prevention. The absence of problem gambling severity group differences in acceptability, further demonstrates broad interest in BetWell. These acceptability and perceived usefulness findings correspond with support from personal budget use literature, whereby such approaches can enhance well-being, autonomy, and perceived control among individuals experiencing mental health problems [65].

BetWell focuses on the financial implications of betting and within our sample, those with greater financial well-being reported higher acceptability and usefulness compared to those with lower financial well-being. Participants within the lowest financial well-being category of ‘Having Trouble’ had notably lower acceptability and usefulness scores than all other groups. This pattern may potentially reflect avoidance, whereby individuals experiencing financial strain may be reluctant to examine their gambling spend and reflect on the implications of this [66]. Although, an important limitation of this finding is the small sample size of participants in the lowest financial well-being group (n=6) relative to the other financial well-being groups. While participants in this group reported lower acceptability than those in the higher financial well-being groups, estimates for this subgroup are less precise and may be disproportionately influenced by individual responses. Consequently, these findings should be interpreted with caution. Further research with a larger and more representative sample of individuals across the full spectrum of financial well-being is needed to determine whether this pattern of results can be replicated.

Participants already using activity statements and tracking their gambling spend indicated greater perceived use of BetWell than those who did not already review or track their betting. Our results are consistent with research indicating that people who use one gambling harm minimization tool are more likely to use others [67]. It suggests that BetWell may meet an existing need within regular bettors and implementation strategies should focus on enhancing engagement by demonstrating this value.

Gambling frequency and the number of accounts held not predicting acceptability or usefulness could be explained by the limited variance within our sample. Most participants reported gambling at least once per week and held multiple accounts which is likely a consequence of the eligibility requirement for study participation. Nevertheless, this finding aligns with Gainsbury et al [15] who reported no associations between harm minimization tool use and gambling frequency nor the number of gambling accounts. Future evaluation would benefit from examining a broader range of gambling frequencies to better determine their impact on BetWell perceptions once the tool becomes accessible to all gambling consumers, allowing for assessment of naturalistic uptake and engagement.

Qualitative feedback is limited by the voluntary nature of the items, however, it provides further insight into user perceptions towards BetWell’s acceptability, usefulness, and usability, and clarifies the quantitative descriptive findings. Participants described the tool as the “best thing I have seen,” reflected on how it contextualized gambling spend relative to income and other expenses, and noted that it improved their understanding of gambling. Participants also identified opportunities to refine BetWell by suggesting that future iterations incorporate interactive elements to encourage engagement, provide additional details on gambling activity, include more challenging quiz content, and ensure that the quiz content is not negatively framed. Participant feedback further highlighted the need to clarify data security to increase their confidence in the tool and improve functionality to automate statement uploads. While the inclusion of interactive elements, challenging and more positively framed quiz content, a greater emphasis on data security, and automating activity statement upload functionality can be incorporated into future BetWell iterations, a more detailed analysis of betting data would require participants to share transaction level data. This is not routinely included in activity statements, nor available for download in a shareable format by most wagering operators.

### Limitations

This exploratory study has several limitations and the findings should be considered in light of these constraints. First, the sample predominantly comprised English-speaking, financially stable, employed males who already accessed their activity statements. As a results, the findings cannot be generalized beyond these demographics, and future research should examine more diverse samples to better understand perceived acceptability and usefulness within the broader gambling population. Second, the study assessed perceived usefulness using a single, author-generated scale designed to capture feature-specific evaluations. As this measure was developed for this study, it did not undergo formal scale development procedures, and we could not establish its content validity. Third, the study may be influenced by sampling bias, as individuals who were especially motivated or interested in gambling-related tools may have been more likely to participate. In addition, the inclusion criterion requiring access to activity statements may have selectively recruited participants with more favorable attitudes towards engaging with such information. These factors may have contributed to more positively biased evaluations of BetWell’s acceptability and usefulness. Finally, a single coder conducted the qualitative analysis, and the study did not implement a comprehensive coding process that would allow assessment of interrater reliability.

### Strengths

The mixed-methods, co-design, and iterative development process used to evaluate and refine BetWell is a strength of the tool design and is consistent with the human-centered design framework. Gaining feedback from target users is an important consideration for intervention design as it assists with identifying ways for improving the tool prior to naturalistic evaluation of tool engagement [40,41]. In our study, participants had the opportunity to engage with a working tool prototype and were free to use it as they would if it were readily available to them, although they were provided with some brief instructions to ensure that they explored all its features. Given that the research setting and recruitment strategy were not ecologically valid, we were unable to draw conclusions regarding feasibility and engagement from this study design. An important next step in the development and evaluation of BetWell is to examine naturalistic patterns of engagement and use.

BetWell’s design incorporates behavioral change technique active ingredients, which are commonly used in digital intervention research, such as Goals and Planning, and Feedback and Monitoring. This is a strength of the tool and its intervention mechanisms. These evidence-based components target awareness and behavior change [68,69] and their inclusion supports future research evaluating BetWell to assess user engagement and tool effectiveness.

### Conclusions and Future Directions

Our preliminary findings provide support for BetWell as acceptable and useful among a sample of regular online gambling consumers. Contrary to BetWell’s prevention-focused intention, the tool was considered more useful among those experiencing gambling problems, demonstrating an additional use case of the tool in assisting people with reflecting on their gambling, which should be explored as a pathway to or adjunct to more intensive intervention. Findings from this study informed subsequent iterations of BetWell, which is now available for use. We incorporated some of the participant feedback by simplifying the activity statement summary visuals, enabling optional forwarding of statements to BetWell user accounts to address limitations of manual uploads, removing the psychoeducational feature to allow further development, and expanding alternative spend comparisons to accommodate a wider range of betting frequencies.

BetWell builds on and extends existing harm minimization tools including mandated activity statements in Australia by amalgamating these in one place to allow cross-operator net results to be readily viewed and reflected upon. Lower perceived acceptability and usefulness by those with lower financial well-being highlights an important area for improvement. Individuals experiencing financial stress are often excluded from budget tools which focus on savings and this group is likely to benefit from greater support with financial decisions including informed betting decisions. As there are several limitations to the study, further investigation in larger and more diverse samples will help clarify whether this pattern is present in more ecologically valid samples of gambling consumers. Nevertheless, using a human centered design framework and iterative development process has provided critical insights to guide the refinement, iteration, and next steps for the development of a digital tool to enhance sustainable online betting.

BetWell is designed to function in Australia as it uses existing mandated customer account statements. This is a unique opportunity for consumers, yet this level of transparency is not broadly available and limited to static pdfs. Following further investigation of the BetWell including its impact on gambling awareness and behavior, implementation strategies involving gambling regulators and operators can be identified. Gambling regulators should consider mandating data portability such that machine-readable, standardized data exports from licensed operators are readily available to customers. This would create the infrastructure to enable cross-operator spend visibility required for consumers to have transparency over their own spend.

## Supplementary material

10.2196/101430 Multimedia Appendix 1 Screengrabs of BetWell’s features and functionality.

10.2196/101430 Checklist 1 CHERRIES checklist.
