# Supplementary material for: Acceptability and Perceived Usefulness of a Digital Gambling Harm Minimization Tool: Cross-Sectional Study
Source: JMIR Form Res. 2026 Aug 7;10:e101430. doi: 10.2196/101430 (PMC13449979; doi:10.2196/101430)
Supplement: Supplementary file 1 [file formative-v10-e101430-s001.docx]

Multimedia Appendix 1.

Screengrabs of BetWell’s features and functionality.

**1. Pre-data check-in**

**2. Aggregated spend dashboard**

Multimedia Appendix 1. (Continued)

**3. Alternative spend comparison**

Multimedia Appendix 1. (Continued)

**4. Psychoeducational quiz comparison**
