# Supplementary material for: Acceptability and Perceived Usefulness of a Digital Gambling Harm Minimization Tool: Cross-Sectional Study
Source: JMIR Form Res. 2026 Aug 7;10:e101430. doi: 10.2196/101430 (PMC13449979; doi:10.2196/101430)
Supplement: Supplementary file 2 [file formative-v10-e101430-s002.docx]

Multimedia Appendix 1.

# Checklist for Reporting Results of Internet E-Surveys (CHERRIES)

| **Checklist Item** | **Explanation** |
| --- | --- |
| Describe survey design | **Describe target population, sample frame. Is the sample a convenience sample? (In “open” surveys this is most likely.)**  Target population: To accurately capture end-user perspectives, we collected responses from gambling consumers who gambling online.  Sampling frame: We employed purposive sampling where participants meeting the specific sampling criteria were eligible to participate in the study. The eligibility criteria considered whether participants were adults, placed bets with at least two major Australian online wagering operators in the previous month and could access to their activity statements to test the tool, could speak, read, and write English, and were receiving treatment related to their gambling. |
| IRB approval | **Mention whether the study has been approved by an IRB.**  The study was approved by the University of Sydney’s Human Research Ethics Committee [Protocol number 2024_HE000747]. |
| Informed consent | **Describe the informed consent process. Where were the participants told the length of time of the survey, which data were stored and where and for how long, who the investigator was, and the purpose of the study?**  The front page of the survey contained a participant information statement which outlined the aims of the project, participation requirements (i.e., eligibility requirements, survey duration), who the study investigators were, data management procedures (i.e., data use and storage), and who to contact for further information. Participants were informed that if they completed the survey, they would receive reimbursement in the form of a $20 e-gift voucher via the market research platform. Participants were informed that by submitting the survey form, they are providing consent to participate in the study. |
| Data protection | **If any personal information was collected or stored, describe what mechanisms were used to protect unauthorized access.**  No personal information was collected.  The survey data was acquired with the use of The University of Sydney Enterprise Qualtrics. All collected data were stored in The University of Sydney’s Enterprise edition of OneDrive, a cloud-based storage service that stores data on secure servers within Australia. Upon completion of the research, data will be stored on the University of Sydney’s Research Data Store, which is a secure, enterprise-grade Network Attached Storage device located within NSW. |
| Development and testing | **State how the survey was developed, including whether the usability and technical functionality of the electronic questionnaire had been tested before fielding the questionnaire.**  The questionnaire was developed by the research team, which comprised of experts in conducting survey research. The survey contained questionnaires which were previously validated based on the literature and also contained some author-generated items which were specific to the study. For example, questionnaire items were constructed which were specific to the Bet Well tool that participants reviewed during the study.  The technical functionality of the survey was tested by the internal team prior to survey distribution via the market research platform. |
| Open survey versus closed survey | **An “open survey” is a survey open for each visitor of a site, while a closed survey is only open to a sample which the investigator knows (password-protected survey).**  The survey was accessible only to a subset of panel members of the market research platform, who were invited to participate in the study. |
| Contact mode | **Indicate whether or not the initial contact with the potential participants was made on the Internet. (Investigators may also send out questionnaires by mail and allow for Web-based data entry.)**  The market research platform contacted individuals via email, which contained a recruitment advert, inviting them to participate in the study. |
| Advertising the survey | **How/where was the survey announced or advertised? Some examples are offline media (newspapers), or online (mailing lists – If yes, which ones?) or banner ads (Where were these banner ads posted and what did they look like?). It is important to know the wording of the announcement as it will heavily influence who chooses to participate. Ideally the survey announcement should be published as an appendix.**  Recruitment adverts were distributed to selected market research panel members via email. The advert contained the following study details:  “Online survey now open  Hi ^name^,  We have an opportunity for you to make a real impact on the products and services you love. Our client is conducting a market research study and would value your opinions and experiences.  If you are interested and want to see if you qualify, please click on the link below. This link is especially for you, ^name^, please do not share this link with anyone else. Click here to start.  Project Information  You were selected for the study. This survey is only open for a limited amount of time. For completing the survey, you will earn points towards a $20 eGift voucher.  This is a survey on understanding how regular bettors may benefit from using a digital resource to help track their betting. Findings from this survey will help refine the resource to be effective and useful for bettors.  This survey will take approximately 20 minutes.  To get started, click on the link below. Even is this survey isn't right for you, you can still earn reward points.  Click here to start.” |
| Web/E-mail | **State the type of e-survey (eg, one posted on a Web site, or one sent out through e-mail). If it is an e-mail survey, were the responses entered manually into a database, or was there an automatic method for capturing responses?**  Responses were captured automatically via Qualtrics. |
| Context | **Describe the Web site (for mailing list/newsgroup) in which the survey was posted. What is the Web site about, who is visiting it, what are visitors normally looking for? Discuss to what degree the content of the Web site could pre-select the sample or influence the results. For example, a survey about vaccination on a anti-immunization Web site will have different results from a Web survey conducted on a government Web site.**  No website was used. Potential respondents were contacted through the market research panel via email containing the survey link. |
| Mandatory/voluntary | **Was it a mandatory survey to be filled in by every visitor who wanted to enter the Web site, or was it a voluntary survey?**  The survey was voluntary. All participants had an option to ignore e-mails. Participants were also informed that they were free to withdraw from the study at any time and should they wish to withdraw, they may stop the survey at any time. |
| Incentives | **Were any incentives offered (eg, monetary, prizes, or non-monetary incentives such as an offer to provide the survey results)?**  Participants were provided with reimbursement for their time in the form of a $20 e-gift voucher via the market research platform. |
| Time/Date | **In what timeframe were the data collected?**  Data collection took place during May and July 2025. |
| Randomization of items or questionnaires | **To prevent biases items can be randomized or alternated.**  Survey items were not randomised. The items were presented in a logical order based on the constructs being measured. For example, questions about website acceptability were asked immediately after participants engaged with the website to support accurate recall.  The response options within one survey item, which required participants to select from a list of motivations for engaging with the website, were randomised to prevent any order effects. |
| Adaptive questioning | **Use adaptive questioning (certain items, or only conditionally displayed based on responses to other items) to reduce number and complexity of the questions.**  Adaptive questioning was used for one survey item which asked participants to indicate whether they track how much money they spend on gambling. If participants select any response from Rarely to Always, they were presented with an additional item that asked them to rate the importance of a list of spend tracking strategies. If participants selected ‘Never’, this item was skipped. |
| Number of Items | **What was the number of questionnaire items per page? The number of items is an important factor for the completion rate.**  Respondents viewed one questionnaire item per webpage. For scales comprising multiple items related to the same construct, those items were presented together on a single page. For example, the 9-items of the PGSI were presented on one page. |
| Number of screens (pages) | **Over how many pages was the questionnaire distributed? The number of items is an important factor for the completion rate.**  25 pages. This includes two introductory pages (participant information statement, eligibility screening) and one debrief statement at the end of the survey. The survey contained 80 items inclusive of the four optional qualitative response items and three adaptive items. |
| Completeness check | **It is technically possible to do consistency or completeness checks before the questionnaire is submitted. Was this done, and if “yes”, how (usually JAVAScript)? An alternative is to check for completeness after the questionnaire has been submitted (and highlight mandatory items). If this has been done, it should be reported. All items should provide a non-response option such as “not applicable” or “rather not say”, and selection of one response option should be enforced.**  All survey items were mandatory; respondents could not proceed without answering them and were prompted to complete any unanswered items on the page. The only exception was the optional open-ended questions on website features. For these, participants received a notification if the response field was left blank but were still able to continue without providing an answer. |
| Review step | **State whether respondents were able to review and change their answers (eg, through a Back button or a Review step which displays a summary of the responses and asks the respondents if they are correct).**  Participants could not review their responses to change them once they proceeded to the next page. |
| Unique site visitor | **If you provide view rates or participation rates, you need to define how you determined a unique visitor. There are different techniques available, based on IP addresses or cookies or both.**  IP addresses and cookies were used to determine unique visitors to the survey. |
| View rate (Ratio of unique survey visitors/unique site visitors) | **Requires counting unique visitors to the first page of the survey, divided by the number of unique site visitors (not page views!). It is not unusual to have view rates of less than 0.1 % if the survey is voluntary.**  The view rate was not reported since all quantitative data was converted to represent pooled results rather than as single individuals. |
| Participation rate (Ratio of unique visitors who agreed to participate/unique first survey page visitors) | **Count the unique number of people who filled in the first survey page (or agreed to participate, for example by checking a checkbox), divided by visitors who visit the first page of the survey (or the informed consents page, if present). This can also be called “recruitment” rate.**  Consent to participate was implied through submission of the survey form; therefore, only completed survey responses were considered providing consent. Participants were informed that they could withdraw from the study prior to completion of the survey, in which case their data will not be included in the analysis. |
| Completion rate (Ratio of users who finished the survey/users who agreed to participate) | **The number of people submitting the last questionnaire page, divided by the number of people who agreed to participate (or submitted the first survey page). This is only relevant if there is a separate “informed consent” page or if the survey goes over several pages. This is a measure for attrition. Note that “completion” can involve leaving questionnaire items blank. This is not a measure for how completely questionnaires were filled in. (If you need a measure for this, use the word “completeness rate”.)**  Completion rate was not reported as consent to participate was implied through submission of the completed survey form. |
| Cookies used | **Indicate whether cookies were used to assign a unique user identifier to each client computer. If so, mention the page on which the cookie was set and read, and how long the cookie was valid. Were duplicate entries avoided by preventing users access to the survey twice; or were duplicate database entries having the same user ID eliminated before analysis? In the latter case, which entries were kept for analysis (eg, the first entry or the most recent)?**  The market research panel used unique user identifiers to prevent respondents from submitting duplicate responses. |
| IP check | **Indicate whether the IP address of the client computer was used to identify potential duplicate entries from the same user. If so, mention the period of time for which no two entries from the same IP address were allowed (eg, 24 hours). Were duplicate entries avoided by preventing users with the same IP address access to the survey twice; or were duplicate database entries having the same IP address within a given period of time eliminated before analysis? If the latter, which entries were kept for analysis (eg, the first entry or the most recent)?**  IP checks were conducted following data collection and during the data cleaning process to identify duplicate responding. |
| Log file analysis | **Indicate whether other techniques to analyze the log file for identification of multiple entries were used. If so, please describe.**  The market research panel created a unique id for the users to prevent and record duplicate entries. For data cleaning purposes, the IP addresses, latitude and longitude coordinates, and market research panel created unique ids were reviewed to identify duplicate responding. |
| Registration | **In “closed” (non-open) surveys, users need to login first and it is easier to prevent duplicate entries from the same user. Describe how this was done. For example, was the survey never displayed a second time once the user had filled it in, or was the username stored together with the survey results and later eliminated? If the latter, which entries were kept for analysis (eg, the first entry or the most recent)?**  The market research panel users were required to have a verified account to access the survey. If a user already completed the survey, their survey link would not allow them to complete the survey again and provide a message to state that they have already completed the survey.  In cases where duplicates were identified based on IP addresses, latitude and longitude coordinates, and the responses were legitimate, the first response was used. For example, there were instances in the study where a panel user contacted the market research panel to suggest that they completed and submitted the form as they were unsure if the form was submitted properly. |
| Handling of incomplete questionnaires | **Were only completed questionnaires analyzed? Were questionnaires which terminated early (where, for example, users did not go through all questionnaire pages) also analyzed?**  Incomplete questionnaires were removed from the dataset as only completed survey responses were considered providing consent to include the data in research. Only completed questionnaires were used in the analysis. |
| Questionnaires submitted with an atypical timestamp | **Some investigators may measure the time people needed to fill in a questionnaire and exclude questionnaires that were submitted too soon. Specify the timeframe that was used as a cut-off point, and describe how this point was determined.**  Survey responses with durations of less than 15 minutes or greater than 60 minutes were excluded from analysis. The minimum expected time for tool engagement was approximately 10 minutes, with an additional 10 minutes estimated for survey completion.  To account for participants who may have uploaded multiple activity statements (each requiring individual upload) and the expected variability in completion times, an upper threshold of 60 minutes was applied. Durations exceeding this limit were considered unlikely to reflect genuine tool use and survey completion and were therefore excluded. |
| Statistical correction | **Indicate whether any methods such as weighting of items or propensity scores have been used to adjust for the non-representative sample; if so, please describe the methods.**  No such methods were used since this was a descriptive project. Analysis methods are described in the corresponding paragraph. |

This checklist has been modified from Eysenbach G. Improving the quality of Web surveys: the Checklist for Reporting Results of Internet E-Surveys (CHERRIES). J Med Internet Res. 2004 Sep 29;6(3):e34 [erratum in J Med Internet Res. 2012; 14(1): e8.]. Article available at [https://www.jmir.org/2004/3/e34/;](https://www.jmir.org/2004/3/e34/%3B) erratum available [https://www.jmir.org/2012/1/e8/.](https://www.jmir.org/2012/1/e8/) Copyright ©Gunther Eysenbach. Originally published in the Journal of Medical Internet Research, 29.9.2004 and 04.01.2012.
